# The Physiological and Pathological Mechanisms of LIN2, LIN7, LIN10 and Their Tripartite Complex

**DOI:** 10.1111/jcmm.70794

**Published:** 2025-08-13

**Authors:** Yangyang Shang, Xinyi Gan, Yue Dang, Jie Liu, Peijun Liu

**Affiliations:** ^1^ Center for Translational Medicine The First Affiliated Hospital of Xi'an Jiaotong University Xi'an Shaanxi China; ^2^ Key Laboratory for Tumor Precision Medicine of Shaanxi Province The First Affiliated Hospital of Xi'an Jiaotong University Xi'an Shaanxi China

**Keywords:** apical‐basal polarity, LIN2, LIN7, LIN10, receptor localisation, synaptic transmission, tumourigenesis

## Abstract

The LIN family represents a set of conserved proteins that are pivotal in the establishment of cell polarity, the development of synapses and signal transduction processes. Its members, polarity proteins LIN2, LIN7 and LIN10, interact with diverse target proteins via the PDZ domain, SH3‐GK tandem domain and PTB domain. Through these interactions, they are actively engaged in the establishment and modulation of apical‐basal polarity. Moreover, LIN2, LIN7 and LIN10, along with their associated complex LIN2/7/10, participate in the physiological phenomena of synaptic transmission and receptor localisation. In addition, from a pathological perspective, LIN2, LIN7 and LIN10 are intricately linked to the genesis and progression of type 2 diabetes, cardiovascular disorders and a wide spectrum of tumours. This review focuses on the polarity proteins LIN2, LIN7, LIN10 and their complex. It summarises the functions of these molecular domains, systematically arranges their regulatory mechanisms in both physiological and pathological contexts and summarises the current state of research on LIN2, LIN7, LIN10 and their complex. The objective is to furnish a robust theoretical foundation for the prospective utilisation of polarity proteins and their complex as cancer markers and therapeutic targets.

**Abbreviations**
AJC: adhesion junction complex
AMPA: α‐amino‐3‐hydroxy‐5‐methylisoxazole‐4‐propionic acid
APBA1: adapter protein X11 alpha
aPKC: atypical protein kinase C
APP: amyloid precursor protein
BLT2: leukotriene B4 receptor type 2
CaMK: Ca^2+^/calmodulin‐dependent protein kinase
CASK: calcium/calmodulin‐dependent serine protein kinase
CDC42: cell division control 42
CRC: colorectal cancer
EGFR: epidermal growth factor receptor
EMT: epithelial mesenchymal transition
GK: guanylate kinase
grnd: grindelwald
HCC: hepatocellular carcinoma
JNK: c‐Jun N‐terminal kinase
MyoV: myosin V
Pals1: LIN7 1 associated protein
Patj: Pals‐associated tight junction protein
PDZ: PSD95/Dlg/ZO1
PIST: Golgi‐associated PDZ and coiled‐coil motif‐containing protein
STXBP1: syntaxin binding protein 1
TAL: Henle's loop

## 1 Background

**FIGURE 1:**
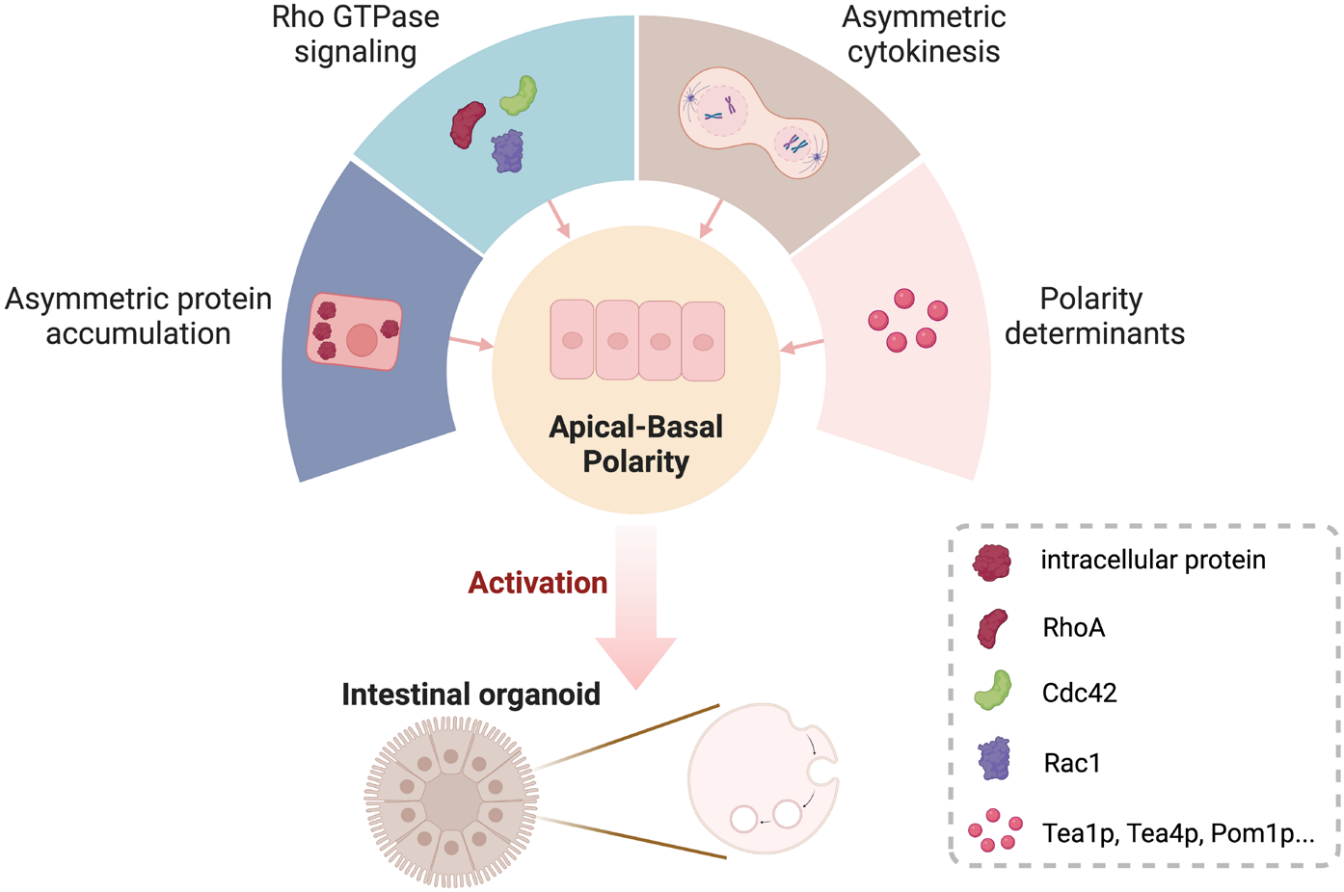
Establishment and function of apical‐basal polarity. Apical‐basal polarity is primarily involved in maintaining epithelial cell morphology and establishing a physiological barrier, as well as specifically targeting and transporting proteins and lipids. The establishment and maintenance of apical‐basal polarity and its regulation are mainly dependent on these four aspects: (1) asymmetric protein accumulation; (2) Rho GTPase signalling; (3) asymmetric cytokinesis; and (4) polarity determinants.

Epithelial cells exhibit polarisation along the cell axis constituting the apical‐basal polarity, which acts in maintaining the morphology and function of epithelial cells, targeting proteins and lipids specifically and performing barrier and transport functions in normal physiology [1, 2]. Apical‐basal polarity is mainly established by three major polarity complexes defined by conserved polarity factors [3, 4, 5]. Additionally, multiple polarity proteins play an indispensable role in the establishment of apical‐basal polarity. The establishment of apical‐basal polarity is primarily governed by asymmetric protein accumulation, Rho GTPase signalling, asymmetric cytokinesis and the involvement of polarity determinants [6, 7, 8, 9] (Figure 1).

Early investigations into apical‐basal polarity were predominantly conducted in *C. elegans* and Drosophila [10, 11]. Polarity proteins LIN2, LIN7 and LIN10 were first identified in *C. elegans* to play a critical role in the localisation of LET‐23 at the basolateral membrane, and when LIN2, LIN7 and LIN10 are mutated, the localisation of LET‐23 is altered to the apical membrane, which is mislocalised [12, 13, 14]. The LIN2/7/10 complex assembles through sequential interactions, LIN10 first recruits LIN2, which then recruits LIN7 to form a stable tripartite structure. The regulation of the LIN2/7/10 complex relies on Golgi apparatus assembly, where LIN10 plays a dominant role in recruiting LIN2 and LIN7 to the Golgi. Its core function is to mediate the targeted transport of LET‐23 EGFR to the basolateral membrane of *C. elegans* vulva precursor cells through this assembly process. In mammals, the complex is also involved in the localisation of polarised membrane proteins, with functions encompassing membrane anchoring, Golgi secretion and transcriptional regulation, thereby exhibiting functional diversity. In neural development, mutations in LIN2 disrupt the assembly of the complex, leading to abnormal localisation of synaptic proteins such as NMDA receptors and causing cognitive deficits. In oncogenesis, abnormal regulation of membrane localisation of receptors like EGFR by the complex enhances the invasive ability of cells [12, 15, 16]. This tripartite complex exhibits remarkable evolutionary conservation [17, 18, 19]. LIN2, LIN7 and LIN10 and their complex play important roles in five aspects: (1) formation and maintenance of basolateral polarised cell structure [20]; (2) regulation of synaptic vesicle exocytosis and synaptic connections [16]; (3) the ability to regulate polarised protein localisation: bind to LET‐23 receptor tyrosine kinase and participate in its basolateral localisation [12, 21]; (4) L27 domain: mediated targeting of NMDA receptors in neurons [17]; (5) PDZ domain: composes and maintains the protein in epithelial cells in binding with the PDZ domains of other transmembrane protein. The binding of the PDZ domain to β‐catenin maintains voltage‐gated calcium channels [12, 22]. This review systematically summarises the structure and functions of the constituent molecules of LIN2, LIN7 and LIN10, and explores their changes under physiological and pathological conditions. By integrating research findings, it consolidates the theoretical foundation for the application of polarity proteins and their complexes in the identification of cancer markers and the discovery of therapeutic targets.

## 2 Structure of LIN2, LIN7 and LIN10

**FIGURE 2:**
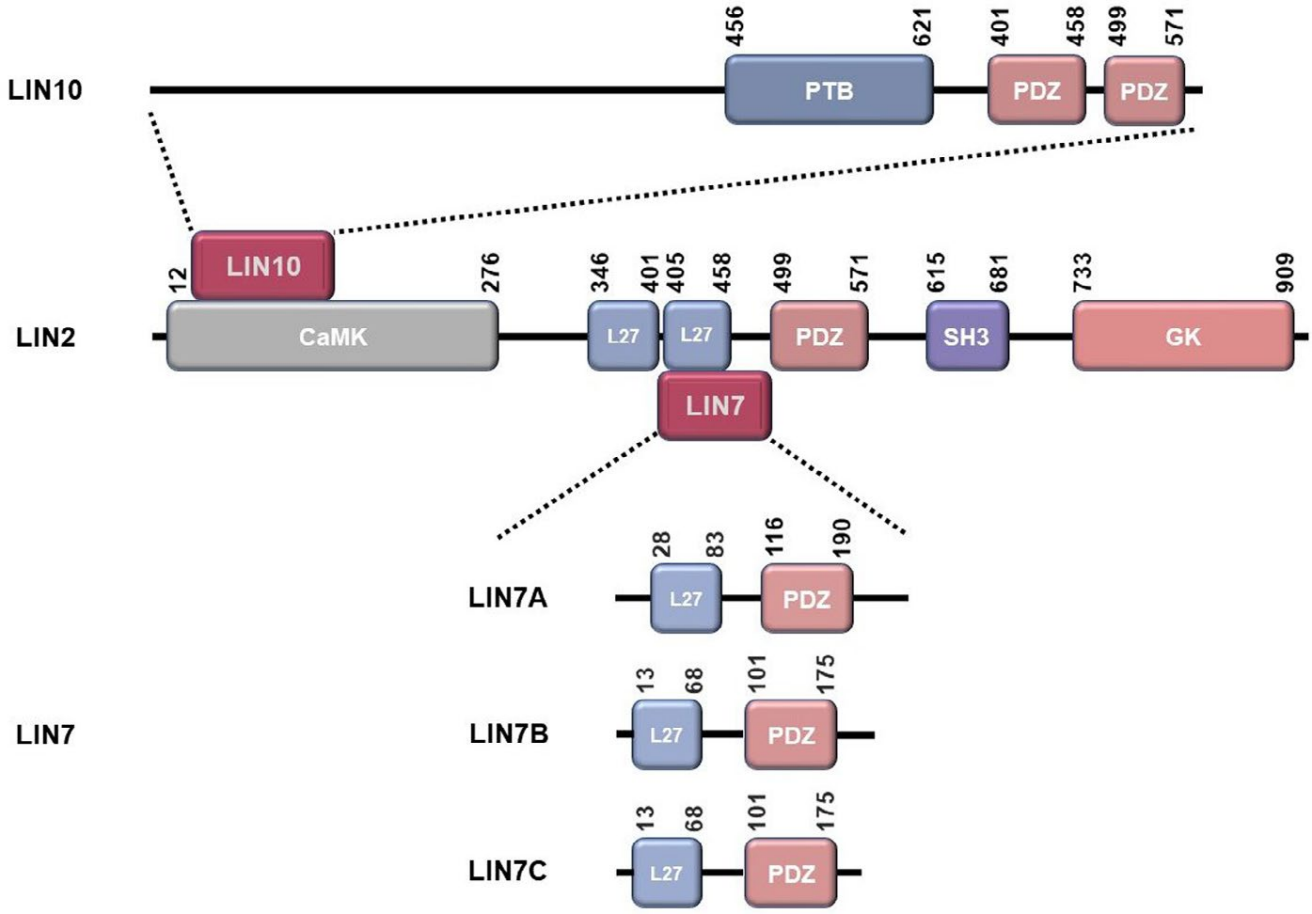
Structure of the LIN2/7/10 complex and its members. In the LIN2/7/10 complex, LIN2 binds both LIN7 and LIN10 to each other, whereas no binding occurs between LIN7 and LIN10. LIN10 binds to the CaMK domain of LIN2 mainly through the N‐terminal unstructured region. LIN7 binds to the L27 domain of LIN2. The polarity complex exhibits striking conservatism.

LIN2, LIN7 and LIN10 correspond to CASK, mLIN7 and MINT1 in mammals [23, 24]. It was shown that in mammals CASK is mainly concentrated at the neuronal synapse and binds to the cell surface scaffolding proteins VELI and MINT1 to form a stable complex through the L27 domain and CaMK domain (Figure 2). MINT1 binds to the CASK‐CaMK domain with a low nanomolar dissociation constant via an extended sequence in the N‐terminal unstructured region, and structurally Ca^2+^/CaM is not involved in regulating the mutual binding of CASK and MINT1.

### 2.1 Structure of LIN2

LIN2, a pluripotency gene localised on chromosome Xp11.4, encodes a calcium/calmodulin‐dependent serine protein kinase belonging to the MAGUK protein family, so it is also referred to as calcium/calmodulin‐dependent serine protein kinase (CASK), CAGH39, FGS4, EC 2.7.11.1, TNRC8, HCASK, CAMGUK MICPCH, MRXSNA and CMG. LIN2 protein is composed of five domains, including SH3, PDZ, L27, Ca^2+^/calmodulin‐dependent protein kinase (CaMK) and guanylate kinase (GK) [25, 26]. LIN2 functions as a conserved multidomain scaffolding protein involved in brain development, synapse formation and establishment of cell polarity by interacting with a variety of proteins. At the early stage of skin injury, LIN2 can co‐localise with Cx43 at the plasma membrane to regulate the distribution of gap junction proteins, thereby promoting wound healing [27]. In the nervous system, its SH3 and PDZ domains act as acetylcholine receptor scaffolds. By forming complexes with FRM‐3 to recruit AChRs, LIN2 mediates synaptic transmission of neurotransmitters. Whether participating in intercellular communication during wound repair in skin cells or regulating receptor aggregation on the postsynaptic membrane in neurons, LIN2 embodies its core role as a scaffold protein in organising signalling complexes across different cellular environments [28]. Mutations in the CaMK domain, an atypical kinase sensitive to Mg^2+^, can lead to the development of pontocerebellar hypoplasia, a disease with a microcephaly phenotype, and a life‐threatening mutation [29]. Distinct from other members of the MAGUK family of proteins, the CaMK domain located at the N‐terminal end of LIN2 has a high degree of sequence unity, and the neurexin tail can be phosphorylated by the LIN2‐CaMK domain in a kinase‐independent form that is not dependent on Mg^2+^ [18]. Amusingly, the CaMK domain located at the N‐terminal end of LIN2 has also been shown to interact with a brain‐specific junction protein known as Caskin 1 [24, 30]. As a member of the MAGUK protein family, calmodulin can regulate the interaction between LIN2 and other scaffolding proteins by binding to the SH3, GK domain of LIN2 in the hooked region [31].

### 2.2 Structure of LIN7

In mammals, the LIN7 family contains three homologous isomers, LIN7A, LIN7B and LIN7C, also named VELI1, 2, 3 or MALS1, 2, 3. Among these homologues, LIN7A and LIN7C are expressed in retinal tissues [32, 33]. LIN7 expression has been detected in renal epithelial cells. The cell‐specific markers were specifically conjugated to antibodies against the three isoforms of LIN7. In mammals, mLin7 is expressed at nectin‐based cell–cell junctions. Nectin is an immunoglobulin‐like intercellular adhesion molecule that participates in the adhesion of epithelial cells in tissues and the formation of tight junctions. The reciprocal binding between nectin and an actin filament‐binding protein named afadin is essential for the correct localisation of mLin7. However, studies have shown that mLin7 does not bind directly to nectin or afadin [34].

#### 2.2.1 Structure of LIN7A

LIN7A is localised on chromosome 12q21.31, which encodes a protein involved in maintaining the asymmetric distribution of cell membrane receptors and channels. LIN7A/MALS1/VELI1 was found to be highly expressed mainly in the glomerulus, Henle's loop (TAL) and distal tubule. The PDZ domain of LIN7A mediates the recruitment of the α2 subunit by heterodimerisation of the β1 subunit to cell–cell contacts in HEK293 cells through binding to the PDZ ligand motif of the α2/β1 complex [35].

#### 2.2.2 Structure of LIN7B

LIN7B is localised on chromosome 19q13.33, which encodes a protein localised in the basolateral plasma membrane with functions involved in neurotransmitter secretion and maintenance of apical‐basal polarity of epithelial cells. LIN7B/MALS2/VELI2 was expressed only in the vasa recta.

#### 2.2.3 Structure of LIN7C

LIN7C is localised on chromosome 11p14.1, also known as FLJ11215, and encodes a protein localised mainly at the cell membrane, with a small amount distributed in the cytoplasm and nucleus. The three LIN7 isoforms are highly similar in mammals, all containing an amino‐terminal L27 domain and an accompanying PDZ domain [36]. LIN7C/MALS3/VELI3 was more abundantly expressed mainly in the collecting duct and distal tubule [37]. LIN7C, a component of the Crumbs complex, participates with Pals1, Patj and Crumbs3 in the composition of the tightly connected ventricular membrane apical [38]. Leukotriene B4 receptor type 2 (BLT2), which is predominantly distributed in epithelial cells, is transported from the Golgi to the plasma membrane to play a barrier role. In this physiological process, BLT2 transport is mainly dependent on LIN7C [39].

### 2.3 Structure of LIN10

LIN10, also known as APBA1 or MINT1, encodes a protein belonging to the MINT1/X11 protein family, which is defined as a PTB/PDZ domain protein, homologous to mammalian Mints [40]. LIN10 is localised on chromosome 9q21.12. The LIN10 protein contains two domains, PDZ and PTB, of which the PDZ domain has PDZ1 and PDZ2. mLin10 is the mammalian homologue of LIN10 and is abundantly present in the trans‐Golgi network. LIN10 is involved in regulating the atypical hypoxia pathway, and when oxygen activates EGL‐9E, one of the isoforms of EGL‐9, LIN10 binds to EGL‐9E and is recruited to endosomes to increase the loop of GLR‐1 AMPARs to endosomes. Under normal oxygen conditions, p38 MAPK enhanced the binding of LIN10 to EGL‐9E [40, 41].

## 3 The Physiological Functions of LIN2, LIN7, LIN10 and LIN2/7/10 Complex

**FIGURE 3:**
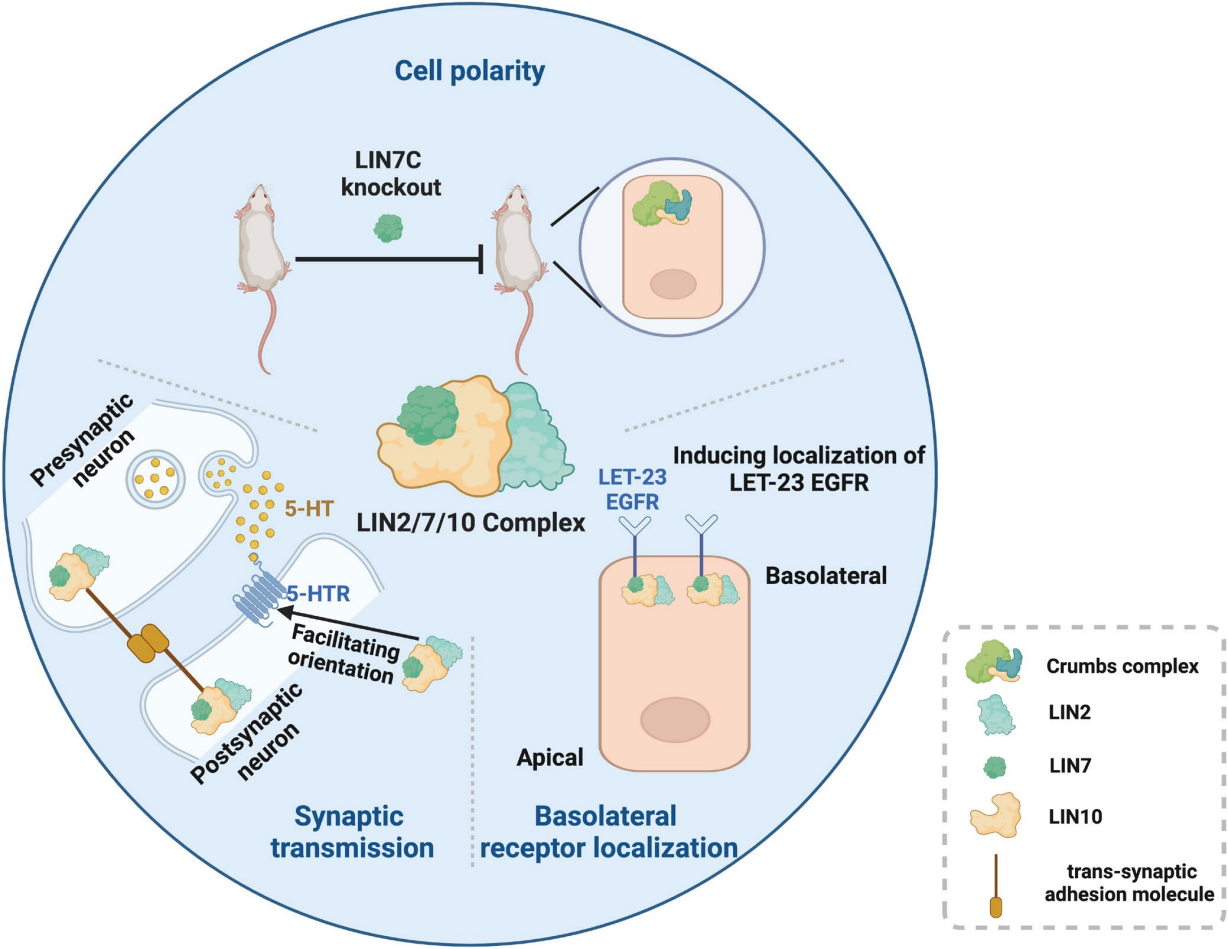
LIN2/7/10 complex and physiological functions. LIN2/7/10 play essential roles in the physiological processes of cell polarity, synaptic transmission and basolateral receptor localisation. Reduction of Crumbs complex can be found in LIN7C‐specific knockout mice, causing renal developmental problems such as tubular dysplasia and interstitial fibrosis in mice. LIN2/7/10 complexes localised on both sides of the synapse anchor trans‐synaptic adhesion molecules to the cytoskeleton on both sides of the synapse and are involved in signal transduction at neuronal synapses. The complex also interacts with 5‐HTR to promote its localisation at the synapse. In addition, the LIN2/7/10 complex is an essential player in the localisation of LET‐23, a member of the EGFR tyrosine kinase family, at the basolateral side.

LIN2, LIN7, LIN10 and their complex perform physiological roles in the establishment of cell polarity, synaptic transmission and basolateral receptor localisation. These physiological functions are summarised in Figure 3.

### 3.1 LIN2, LIN7, LIN10 and LIN2/7/10 Complex Regulate Cell Polarity

Cell polarity is widespread in plant and animal cells and microbial cells, and is the fundamental reason for the formation of biodiversity. Through polarisation, cells are able to sense and trigger timely responses from neighbouring cells and the surrounding microenvironment [42]. The regulation of cell polarity is dependent on the assembly of supramolecular complexes in multidomain scaffolding proteins. The complex composed of LIN2, LIN7 and LIN10 is essential for the maintenance of cell polarity. The LIN2/7/10 complex does not rely on its four PDZ domains, SH3‐GK tandem or PTB domain for complex assembly. Instead, these domains enable the complex to bind diverse target proteins and regulate cellular functions. As such, the LIN2/7/10 complex acts as a central regulatory hub for establishing and maintaining cell polarity [18].

LIN7 protein family members and LIN2 can also team up with the L27 domain of DLG proteins to become a complex that maintains cell polarity and mediates intercellular signalling [43]. LIN7 has been reported to form a complex with E‐cadherin and β‐catenin in epithelial cells and neurons, which are involved in the recruitment of LIN7 during the establishment of cell polarity to the intercellular junctions mediated by cadherin [44]. Renal tubular dysplasia, renal interstitial fibrosis and cystic degeneration caused by defective polarisation of renal tubular epithelial cells of mesenchymal origin were revealed in the kidneys of LIN7C‐specific knockout mice [45]. In addition, three scaffolding proteins, mammalian homologue‐2 of LIN7 (LIN7B), Pals‐associated tight junction protein (Patj) and LIN7 1 associated protein (Pals1), together form a complex that regulates apical polarity by crosstalk between the LIN2 and LIN7 domains [46]. Thus, the LIN2/7/10 complex and its members play a critical role in the maintenance and regulation of cell polarity.

### 3.2 LIN2, LIN7, LIN10 and LIN2/7/10 Complex Participate in Synaptic Transmission

Synapses are the basic units of neuronal signal processing in the brain, and the basis for brain function in mammals during growth depends on the formation of synapses and the transmission and dynamic response to stimulus signals [47]. The LIN2/7/10 complex of PDZ proteins is distributed on both sides of the synapse, linking trans‐synaptic adhesion molecules to the cytoskeleton to participate in neuronal signalling. Intriguingly, when the LIN2/7/10 complex component is mutated, neurons exhibit significant presynaptic defects. It has been found that liprin‐α is a major component of the purified LIN7 protein complex in the brain and that the size and morphology of the active zone are determined by liprin‐α, suggesting that the presynaptic release mechanism is linked to the active zone through this complex, thereby regulating neurotransmitter release [48, 49]. A glutamate receptor that plays an influential role in memory and learning and has synaptic plasticity is known as NMDAR, and the NMDA‐type glutamate receptor NR2 can be specifically recognised by the motor protein KIF17 by binding to the scaffolding complex LIN2/7/10 and then transporting its subunit, the NR2B vesicle, for localisation to fixed dendrites [50]. Moreover, the complex was shown to interact with 5‐HT(2C) receptors in vivo to function as a facilitator of 5‐HT(2C) receptor synaptic localisation [51]. LIN7B was evidenced to interact with Rhotekin, an effector of Rho, in vitro and in vivo to form a complex. Rho signalling regulates the interaction between LIN7B and Rhotekin. It can also be hypothesised that Rhotekin may be involved in the regulation of the LIN2/7/10 complex in the synapse, and the related mechanisms need to be explored in further studies. In addition, PIST was proved to be involved in synaptic transport by binding with Rhotekin to each other. This demonstrates that synaptic transport functions such as neurotransmitter release may depend on the competitive binding of PIST and LIN7 to Rhotekin [52]. Synaptic plasticity of AMPA‐type glutamate receptors is critically dependent on the membrane‐regulated translocation of the receptor. In the transport of AMPA receptors, one of the glutamate receptors, in the synapse, mLin10 is involved in the transport of AMPA receptors through the PDZ domain by interacting directly with glutamate receptors [53]. LIN10 is recruited by RAB‐6.2 and interacts with the GTP‐bound form of RAB‐6.2. Then LIN10, RAB‐6.2 and the retromer complex cooperate to maintain the stability of synaptic abundance by retrograde recycling of the AMPAR subunit GLR‐1 from dendrites to the Golgi apparatus [54]. Therefore, the LIN2/7/10 complex may be a crucial molecule in the synaptic transmission process.

### 3.3 LIN2, LIN7, LIN10 and LIN2/7/10 Complex Are Involved in Regulating Basolateral Receptor Localisation

The activity of the intracellular signalling pathway activated by the binding of epidermal growth factor receptor (EGFR) to its ligand depends on the localisation of EGFR in polarised epithelial cells [55]. The structural dimer LET‐23 is a member of the EGFR tyrosine kinase family in the nematode Hidradenitis elegans [56, 57]. LET‐23 is predominantly localised to the basement membrane of polarised vulvar epithelial cells, and a complex composed of LIN2, LIN7 and LIN10 plays an instrumental role in the basolateral localisation of LET‐23 [12, 58]. The investigators found that the complex co‐localised with LET‐23 EGFR in cytoplasmic puncta and partially overlapping Golgi bodies, and that LIN7 and LET‐23 EGFR co‐localised in the basement membrane [15, 59]. More importantly, direct binding of LIN7 to LET‐23 is necessary for proper localisation of LET‐23. Overexpression of LET‐23 can also partially redeem the vulva‐free phenotype caused by LIN2 and LIN7 mutants [60]. LIN10 participates in mediating basolateral and postsynaptic localisation pathways and has a crucial role in the epithelial and neuronal localisation of GLR‐1 [61]. LIN10 binds to amyloid precursor protein (APP) through the PTB domain and may significantly inhibit the conversion of amyloid precursor protein to amyloid β (Aβ) peptide by modifying the localisation of APP; thus, the LIN2/7/10 complex may play an influential role in neuronal APP transport and processing [62, 63, 64]. Furthermore, mammalian LIN7 coordinates basolateral targeting function through the interaction of the L27 domain with the homologous L27 domain of LIN2 [65].

## 4 The Pathological Functions of LIN2, LIN7 and LIN10

### 4.1 LIN2, LIN7 and LIN10 Impact Type 2 Diabetes Mellitus

Type 2 diabetes mellitus (T2DM) is a chronic metabolic disease. The onset of this disease is usually caused by insulin resistance and pancreatic islet β‐cell defects [66]. As the disease progresses, the ability of pancreatic islet β‐cells in patients with T2DM to secrete insulin decreases. The secretion of insulin becomes insufficient, and the blood glucose level rises more significantly, leading to the aggravation of symptoms [67]. LIN2, LIN7 and LIN10 play roles in the secretion and regulation of insulin. Some studies have shown that the LIN2–LIN10 complex regulates the transport of Munc18‐1 to the cell membrane, thus promoting insulin secretion, and this process is extremely important. Among them, LIN2 may act as a sensor, regulator and organiser, receiving signals and activating relevant pathways. LIN10, on the other hand, serves as a regulator and a mediator. The two work in concert to jointly promote insulin secretion [68]. During the process of insulin secretion, LIN2 forms a ternary complex with LIN10 and STXBP1. By enhancing the interaction among them and mediating their transport to the plasma membrane, it promotes insulin secretion. High fatty acid stimulation reduces LIN2 expression, impairs its binding to LIN10 and its function in mediating the membrane‐targeted transport of LIN10/STXBP1, and disrupts the insulin granule exocytotic molecular axis mediated by the LIN2/LIN10/STXBP1 tripartite complex, ultimately inhibiting insulin secretion [69]. Knocking out LIN2 in mouse pancreatic islet β‐cells interferes with the transport or anchoring of insulin granules to the cell membrane, inhibits glucose‐stimulated insulin secretion and increases the blood glucose level [70]. Interestingly, the inflammatory factor IL‐1β can activate DNA methyltransferase through the iNOS pathway, causing hypermethylation in the promoter region of Cask, reducing the expression of LIN2 and then inducing the insulin secretion dysfunction of pancreatic islet β‐cells [71]. LIN2 is involved in the insulin secretion dysfunction of pancreatic islet β‐cells induced by high glucose toxicity. High glucose down‐regulates the level of LIN2, and the overexpression of LIN2 can partially improve the insulin secretion defect of cells cultured in high glucose [72]. A decrease in LIN2 expression inhibits the glucose‐stimulated insulin secretion function of INS‐1 cells induced by Ex‐4, indicating that it is involved in the insulin secretion mediated by Ex‐4. Moreover, Ex‐4 can promote the expression of LIN2 at both the transcriptional and protein levels through the cAMP/PKA pathway [73]. Han et al. found that FOXO1 mediates the dysfunction of pancreatic islet β‐cells by down‐regulating the expression of LIN2, affecting the anchoring and exocytosis of insulin granules to the cell membrane. LIN2 may regulate insulin secretion by binding to other secretion‐related cytoskeletal proteins, reducing the obstruction of F‐actin to the anchoring and fusion of insulin granules [74]. Insulin receptor substrate p53 (IRSp53) is crucial in insulin secretion. The key region for phosphorylation in the insulin‐dependent pathway is located between the N‐terminal IMD and the central SH3 domain. Tyrosine 310 is an essential site. The N‐terminal IMD is important for efficient phosphorylation and may function through dimerisation. Its phosphorylation state may affect the cellular activities related to insulin secretion [75]. IRSp53 is an adaptor protein at the membrane‐actin interface, which can link membrane deformation to F‐actin polymerisation and is key to the formation of filopodia. LIN7 can regulate IRSp53. The binding of the two is important for the formation of filopodia and neurites, and there is an interaction between them in cytoskeleton‐related activities. This provides a theoretical basis for them to regulate cytoskeleton reorganisation in pancreatic islet β‐cells and affect the transport and fusion of insulin secretion granules [76]. In addition, some studies have indicated that LIN7 can mediate the recruitment of IRSp53 to tight junctions, regulate its localisation and function. The synergistic effect of the two may be related to the regulation of the cytoskeleton during the transport and fusion of insulin secretion granules in pancreatic islet β‐cells, and abnormal insulin secretion in pancreatic islet β‐cells is closely related to the onset of diabetes [77]. LIN10 binds to Neurexin via its PDZ domain to form a complex, which recruits proteins such as LIN2 and LIN7 for plasma membrane anchoring. This complex regulates Granuphilin‐mediated insulin vesicle docking and Munc18‐1‐dependent SNARE complex assembly, and dynamically modulates vesicle fusion through Ca^2+^‐activated LIN2 phosphorylation upon glucose stimulation, thereby achieving precise regulation of insulin vesicle exocytosis. However, in the case of blood glucose fluctuations, the expression level of LIN10 decreases. This not only causes problems in vesicle docking but also hinders the activation of the SNARE complex, a key protein complex for cell exocytosis, interrupts the molecular communication between cells and consequently leads to insufficient insulin exocytosis [78, 79].

### 4.2 LIN2, LIN7 and LIN10 in Cardiovascular Diseases

The inhibition or loss of LIN2 is an important cause of cardiovascular diseases, and it is a molecular target worthy of attention in cardiology research. In both human and mouse hearts, LIN2 plays a role in regulating the activity of Ca/calmodulin‐dependent kinase II (CaMKII), and there is a close interaction between the two. When under pressure overload, the left ventricular ejection fraction of LIN2 knockout mice decreases earlier compared to that of normal mice. This is because the absence of LIN2 enhances the activity of CaMKII in mice, specifically manifested as an increase in T286 phosphorylation and a decrease in T305 phosphorylation. Eventually, it leads to systolic dysfunction, causing an increase in the mortality rate of mice under pressure overload and also increasing the susceptibility to ventricular arrhythmias. In contrast, increasing the expression of LIN2 can inhibit the activity of CaMKII, thereby improving calcium handling. For example, GLP1 receptor agonists can stimulate the expression of LIN2, thus exerting a protective effect on the heart [80]. LIN2 regulates the anterograde transport of the Nav1.5 channel through its L27B and HOOK domains and interacts with the dystrophin‐glycoprotein complex via the HOOK domain. The L27B and GUK domains regulate the functional expression of the NaV1.5 channel on the myocardial cell membrane, affecting the sodium current. LIN2 participates in the construction of the macromolecular complex scaffold through the HOOK domain. The decreased expression of LIN2 in the remodelled myocardium may affect the structural organisation of the sarcomere and the exocytosis of the Nav1.5 channel, which is related to heart diseases such as cardiac anisotropy and sodium channel‐related arrhythmias [81, 82]. In addition, LIN2 can bind to the C‐terminus of the Kir2.2 channel. It is recruited to the C‐terminus of the channel through interaction with SAP97 or LIN7 and forms a stable complex together with SAP97 and others, being associated with the Kir2 channel in the brain and heart. LIN2 co‐localises with SAP97 at the Kir2.2 and Kir2.3 channels, and its dominant negative construct will lead to the mislocalisation of the Kir2.2 channel in the cell. LIN2 is widely expressed in the heart and may affect the function of the Kir2 channel. Its abnormal function or expression changes may affect the cardiac electrophysiological process, thereby leading to the occurrence of heart diseases [83]. Currently, there are relatively few studies on the relationship between LIN7, LIN10 and cardiovascular diseases. However, considering the important role of the complex composed of LIN2, LIN7 and LIN10 in the process of cell biology, it is still necessary to conduct in‐depth research in the future on the potential functions of LIN7 and LIN10 in the cardiovascular system and their relationships with cardiovascular diseases.

### 4.3 LIN2, LIN7 and LIN10 in Tumourigenesis

**FIGURE 4:**
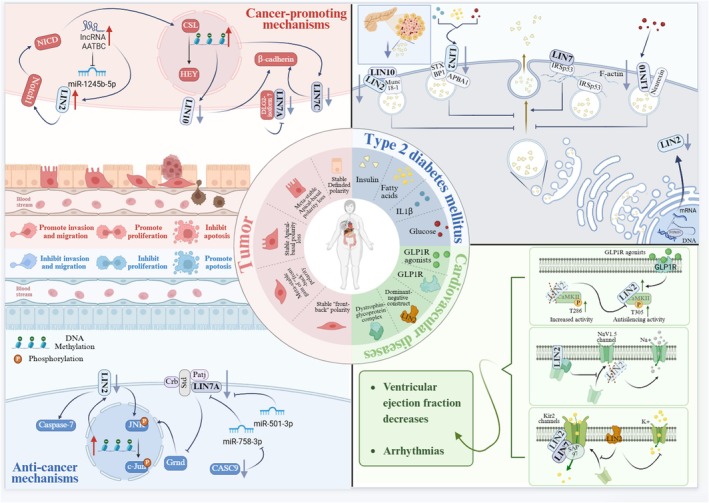
LIN2, LIN7, LIN10 and signalling regulatory mechanisms. LIN2, LIN7 and LIN10 regulate the occurrence and progression of type 2 diabetes by binding to distinct molecules to influence insulin production and transmission. LIN2 and LIN7 are implicated in the development of cardiovascular diseases through modulating the transport of various pathways and channels. LIN2, LIN7 and LIN10 are involved in tumour development through various pathways. LIN2 acts as a pro‐oncogenic molecule and regulates downstream target molecules through the Notch pathway and JNK pathway, reducing apoptosis and promoting malignant progression of cancer cells. LIN7A acts as a downstream target molecule of various microRNAs and plays a role as a pro‐oncogenic or an inhibitory molecule in the development of cancer. Methylation‐induced reduction in the expression of LIN10 and hypermethylation‐induced reduction in the expression of LIN7C can promote the expression of β‐cadherin, which promotes cancer invasion and migration.

Loss of cell polarity is commonly accompanied by abnormal cell morphogenesis, which further leads to diminished cell adhesion and excessive proliferation, which may contribute to the development of several tumours [84, 85]. It has been observed that when proteins that constitute epithelial cell polarity are dysregulated, the loss of epithelial cell polarity leads to the development of invasive tumour metastasis [86, 87, 88]. A portion of polarity proteins that act as tumour suppressor molecules, when intense oncogenic signals are targeted to disrupt these polarity proteins, the resulting polarity defects induced by polarity protein disruption may act in concert with the oncogenic signals to lead to tumour development [89] (Figure 4).

Interestingly, Wu et al. [18] found that a variety of LIN2 mutations in brain diseases and cancer are associated with specific structural abnormalities in the polarity proteins LIN2 and LIN10. High expression of LIN2 in pancreatic adenocarcinoma cell lines was strongly related to poor prognosis in patients with pancreatic adenocarcinoma, whereas in cholangiocarcinoma, LIN2‐negative expression was associated with significantly decreased survival and prognosis compared to LIN2‐expression‐positive patients [90, 91]. The PDZ domain of LIN2 can target binding to CD98, a negative prognostic marker for human glioblastoma cells. Constructing a chimera of the PDZ domain of LIN2 with the ribosome inactivating protein Saporin and enhancing the activity of this chimera as the number of PDZ domains increases, can effectively increase cytotoxicity and apoptosis in human glioblastoma cells, GL15 and U87 [92]. High methylation of LIN10 has also been shown to be closely associated with malignant progression of tumours. Consequently, these findings highlight the intimate association between the polarity complex and tumourigenesis (Table 1).

**TABLE 1:** Expression of LIN2, LIN7 and LIN10 in different cancers and their upstream/downstream effectors.

Name	Expression	Cancer types examined	Upstream/downstream effectors of the pathway	Cell line	References
LIN2	Upregulated	Pancreatic adenocarcinoma	Notch pathway	Notch, CFPAC‐1	[90, 93]
	Upregulated	Hepatocellular carcinoma	Caspase‐7, c‐Jun N‐terminal kinase (JNK) pathway	SMMC‐7721, SK‐Hep‐1 and SMMC‐7721‐sora	[94]
	Upregulated	Prostate cancer	miR‐1245b‐5p	PC3, LNCaP	[95]
	Upregulated	Oesophageal carcinoma	Reelin		[96]
	Deregulated	Cholangiocarcinoma			[91]
	Deregulated	Glioblastoma	Ribosome inactivating protein Saporin, CD98	GL15, U87	[92]
LIN7A	Upregulated	Ovarian cancer	CASC9/miR‐758‐3p/LIN7A axis	SKOV3, A2780	[97]
	Upregulated	Hepatocellular carcinoma	miR‐501‐3p	HCCLM3, PLC/PRF/5	[98]
	Upregulated	Breast carcinomas	MEK/ERK and PI3K/AKT pathway	MCF10A, CAMA‐1 and MDA‐MB‐231	[99]
	Deregulated	Neuroblastoma	DLG2‐isoform 7	SKNAS	[43]
LIN7C	Deregulated	Squamous cell carcinoma	Hypermethylation	Ca9‐22, HSC‐2, HSC‐3 and HSC‐4	[100]
LIN10	Deregulated	Colorectal cancer	Methylation		[101, 102, 103]
	Deregulated	Hepatocellular carcinoma	Methylation		[104]

#### 4.3.1 LIN2, LIN7 and LIN10 Regulate Proliferation in Cancers

A crucial factor in the unrestricted proliferation of tumours is the loss of epithelial polarity [86, 88]. The uncontrolled proliferation that occurs in Drosophila is usually caused by loss of epithelial cell polarity due to loss of function or mutations in various polarity proteins [105]. It has been shown that the polarity protein CASK is significantly upregulated in pancreatic cancer tissues, and silencing of CASK can attenuate the unrestricted proliferation of tumour cells by inhibiting the Notch pathway in pancreatic cancer cells [90, 93]. Furthermore, some investigators have identified that in Drosophila, the grindelwald (grnd) gene encoding a transmembrane protein. The cell polarity determining gene Crb co‐localises with grnd, and the altered activity of Crb and the response of downstream JNK signalling are mainly connected through the interaction of grnd with VELI, which leads to loss of polarity and uncontrolled proliferation of tumour cells [106]. The long noncoding RNA cancer susceptibility 9 called CASC9 is significantly highly expressed in ovarian cancer tissues and cells and correlates with poor prognosis in ovarian cancer patients. Hu et al. found that CASC9 and miR‐758‐3p competitively bind to downstream LIN7A, and that high CASC9 expression can effectively inhibit miR‐758‐3p expression and promote the increase of LIN7A level. In turn, the inhibitory effect on ovarian cancer progression following CASC9 reduction could be reversed by LIN7A overexpression. The CASC9/miR‐758‐3p/LIN7A axis has been shown to be involved in ovarian cancer progression, accelerating tumour proliferation in vivo [97]. Normal colorectal epithelium is generally not methylated, and CpG island methylation, as one of the pathogenic mechanisms of colorectal cancer, is clinically important for the diagnosis and treatment of colorectal cancer. Many previous studies have examined the methylation status of related genes in non‐tumour tissues and colorectal cancer tissues using methylation‐specific polymerase chain reaction technology and found that the genes with significantly increased methylation incidence included LIN10. Elevated levels of methylation of LIN10 have been shown to be closely associated with the development of colorectal cancer (CRC) and hepatocellular carcinoma (HCC), as well as with low survival rates of patients [101, 102, 103]. In addition, hepatocellular carcinoma revealed frequent methylation of all the genes tested compared to normal liver tissue. Among them, the frequency and density of methylation were higher in hepatitis C virus‐associated hepatocellular carcinomas compared with virus‐negative hepatocellular carcinomas, suggesting that viral infection caused by hepatitis C can promote the methylation process [104].

#### 4.3.2 LIN2, LIN7 and LIN10 Regulate Apoptosis and Autophagy in Cancers

Loss of apical‐basal polarity tends to affect the regulation of apoptosis in diverse cancers [107, 108, 109]. In neuroblastoma, the protein encoded by DLG2‐isoform 7 positively regulates and increases the expression of LIN7A by binding to LIN7A, which significantly reduces the proliferative viability of neuroblastoma cells and increases apoptosis of tumour cells [43]. Additionally, hypomethylation‐induced LIN2 expression has been shown to correlate with sorafenib resistance and poor prognosis in hepatocellular carcinoma patients. Down‐regulation of LIN2 can promote apoptosis and autophagy in hepatocellular carcinoma through activation of the JNK pathway, which strengthens the effects of sorafenib [94]. Thus, polarity proteins can induce apoptosis or autophagy in cancer cells by being regulated by upstream molecules or epigenetically modified, thereby increasing patient survival and improving prognosis.

#### 4.3.3 LIN2, LIN7 and LIN10 Regulate Metastasis in Cancers

Metastasis is a hallmark of malignancy, and numerous studies have shown that epithelial cells can acquire invasiveness and metastasis after undergoing transformation from an epithelial to a mesenchymal phenotype. Therefore, epithelial mesenchymal transition (EMT) is one of the important steps in the malignant progression of tumours. A pivotal step in the progression of metastatic cancer is the loss of apical‐basal polarity. After obtaining EMT, the shape of the cell is transformed into an elongated form, at which point the polarity of the cell is referred to as front‐back polarity. Core polarity proteins have been shown to be crucial molecules in the process of constituting and maintaining the cell adhesion junction complex (AJC). Loss of polarity proteins can disrupt cell adhesion by altering epithelial cell organisation, thereby promoting cancer invasion and metastasis [84, 89, 110, 111].

Metastasis has been implicated in pancreatic, breast and prostate cancers caused by deletion or expression of polarity proteins [112, 113, 114]. The lncRNA AATBC binds miR‐1245b‐5p as an oncogene and promotes malignant progression of prostate cancer through the miR‐1245b‐5p/LIN2 axis, and the downstream molecule LIN2 plays a pro‐oncogenic role in prostate cancer progression [95]. LIN2 and its target gene Reelin are obviously high‐expressed in human oesophageal cancer, and the upregulation of Reelin is caused by the overexpression of LIN2, which further participates in oesophageal cancer development by causing signalling pathway disorders [96]. It was found that the expression of LIN2 and Cx43 separately can inhibit cell migration, but when they are co‐expressed, the inhibition of cell migration can be eliminated [27]. In hepatocellular carcinoma, miR‐501‐3p was targeted to inhibit HCC metastasis and progression through LIN7A, a direct functional target [98]. Overexpression of LIN7A disrupts cell polarity and, via PDZ domain‐mediated interactions, activates signalling pathways such as MEK/ERK and PI3K/AKT as well as receptors including INSULIN R and AXL, thereby enhancing the proliferative and invasive capacities of breast cancer cells and contributing to carcinogenesis [99]. Additionally, hypermethylation of the CpG island region of LIN7C in oral squamous cell carcinoma resulted in down‐regulation of expression, whereas overexpression of LIN7C effectively inhibited multi‐organ metastasis in immunodeficient mice [100].

## 5 Summary and Perspective

This review delves deeply into the physiological and pathological functions of the polarity proteins LIN2, LIN7, LIN10 and their complex. Through the analysis and summarisation of the structural domains and physiological functions of these molecules, we have found that LIN2, LIN7, LIN10 and their complex play important roles in the establishment and maintenance of apical‐basal polarity. They are also involved in two physiological processes: synaptic transmission and receptor localisation. In addition, LIN2, LIN7 and LIN10 are associated with the pathological processes of type 2 diabetes mellitus and cardiovascular diseases. Meanwhile, they regulate the proliferation, apoptosis and metastasis of cancer cells through various pathways.

At present, partial research reports have been published on the physiological and pathological functions of LIN2, LIN7 and LIN10 individually. However, the roles of LIN2, LIN7, LIN10 and their complexes in the progression of type 2 diabetes, cardiovascular diseases and cancer require further in‐depth investigation. Additionally, current explorations of LIN2, LIN7, LIN10 and their complexes remain at the theoretical level, with no targeted therapeutic drugs having been explored or tested, and theoretical achievements still needing further clinical translation. In summary, this review deepens our understanding of the physiological and pathological roles of the polarity proteins LIN2, LIN7, LIN10 and their complexes, providing an important reference for exploring their applications in disease treatment and promoting the integration of basic research with clinical practice.

## Author Contributions

**Yangyang Shang:** conceptualization (lead), data curation (lead), writing – original draft (lead). **Xinyi Gan:** conceptualization (equal), data curation (equal), writing – original draft (equal). **Yue Dang:** conceptualization (equal), data curation (equal), writing – original draft (equal). **Jie Liu:** conceptualization (equal), data curation (equal), writing – original draft (equal). **Peijun Liu:** conceptualization (lead), supervision (lead), writing – review and editing (lead).

## Ethics Statement

The authors have nothing to report.

## Consent

The authors have nothing to report.

## Conflicts of Interest

The authors declare no conflicts of interest.

## Data Availability

No datasets were generated or analysed during the current study.
